# Supplementary material for: Post-traumatic stress disorder symptoms among healthcare workers during the COVID-19 pandemic: Analysis of the HERO Registry
Source: PLoS One. 2023 Nov 9;18(11):e0293392. doi: 10.1371/journal.pone.0293392 (PMC10635468; doi:10.1371/journal.pone.0293392)
Supplement: Attachment — Submitted filename: 24 April, 2023 Comments on the manuscript PLOS One.pdf [file pone.0293392.s001.pdf]

24 April 2023

Thank you for the opportunity to review the manuscript **"Post- Traumatic Stress Disorder symptoms among healthcare workers during the COVID-19 pandemic: Analysis of the HERO registry**". It was interesting to go through the manuscript and to know that this was an outcome of the Patient Centered Outcome Research initiatives. The authors attempt to assess PTSD symptoms among healthcare workers during the COVID-19 pandemic and investigate association with demographic and other factors. Overall, the manuscript is well-written.

However, I have following comments in regard to various sections of the manuscript, and I suggest the authors to consider them in revising their paper. Please consider them as positive feedback and note that I am not an expert in statistical methods.

Title: The authors could reflect on the design of the study in the title itself besides mentioning the registry analysis.

Abstract: Please consider removing the exact values in odd ratios. Providing statements of your findings should suffice in the results.

Introduction: I suggest the authors to give a brief overview on why they think COVID-19 can be equated with Trauma definition for PTSD before proceeding on to literature depicting relation between PTSD and COVID-19.

The authors mention two objectives. When they say burden of PTSD symptoms, what do they mean? It is understandable that persistent stressor like COVID-19 will lead to an increase in levels of stress for most populations, including HCWs. How do they estimate that these symptoms are PTSD symptoms and not anxiety and/ or depression? When they say elevated level of PTSD symptoms, what is the baseline against which they measure the symptoms?

Methods:

What were the eligibility criteria except for being HCWs? Were there any duration criteria for symptoms mentioned?

I understand this was a voluntary registry- based study. However, Regarding sampling…was there any justification for sample size? How many invitations were sent? What was the response rate? What about missing data? Were there any outliers? Also, mention on the design of the study.

The authors mentioned cohort in one instance consisting of HCWs, their family members and members of the community. What was the relevance of this and why is it termed as cohort? Please clarify.

Survey tools

DSM- 5 PTSD screening survey: I suggest the authors to briefly mention regarding this instrument and list its psychometric properties in the given context. The questions in this survey appear vague and threshold of 3 could be met by any depressed or anxious individual as well. Please comment on this statement.

Statistical analysis

I suggest the authors to comment on the distribution of the variables of interest. I see that median has been reported for age, and I assume that it is skewed. Please provide details regarding other variables. Also, please cite the reference for PTSD threshold as 3 and above. Regarding unadjusted model, which statistical test was used? Were assumptions met for multivariate logistic regression? Please provide rationale for inclusion of variables in adjusted models.

Discussion

Discussion needs rewriting. In regard to first objective, how do the authors conclude that there is high level of PTSD symptoms in all levels of HCWs. Please justify.

In line 177- 178, the authors conclude with the statement that excessive stress and loss of resilience occurs to the HCWs during the pandemic. Please clarify on how raised PTSD symptoms means loss of resilience.

Discussion on the association between gender and PTSD is not enough since there is abundant literature on this. Most of the discussion appears generic on relation between psychological sequalae and gender. Please be more specific to in relation to PTSD. Same goes for association with nurses compared to physicians. With age, the evidence is mixed. Please discuss in light of those findings.

Limitations

The authors have listed few limitations. I believe this study has more limitations and biases such as using only screening questionnaire, not ruling out depression or anxiety or even burnout, which are quite common in this scenario. There could be many other factors at play which have not been considered in the given study.

I also suggest the authors to highlight the strengths of this study despite the limitations.

I wish the authors all the best.
