# Supplementary material for: Post-traumatic stress disorder symptoms among healthcare workers during the COVID-19 pandemic: Analysis of the HERO Registry
Source: PLoS One. 2023 Nov 9;18(11):e0293392. doi: 10.1371/journal.pone.0293392 (PMC10635468; doi:10.1371/journal.pone.0293392)
Supplement: Attachment — Submitted filename: PLOSONE Response to Reviewers_September29_Final.docx [file pone.0293392.s003.docx]

September 29^th^, 2023

PONE-D-23-06322R1

**Post-Traumatic Stress Disorder symptoms among healthcare workers during the COVID-19 pandemic: Analysis of the HERO registry**

Dear Vincenzo De Luca,

Thank you very much for the supportive review of our manuscript. We appreciate the additional helpful suggestions from the editor and reviewers and have made the following modifications to improve the presentation of our analysis. We believe the manuscript is greatly improved as a result of these changes.

Again, we appreciate PLOS ONE’s support in the investigation of the importance of PTSD symptoms among healthcare workers during the COVID-19 Pandemic and believe this work will be of great interest to your readership.

All reviewer comments are listed in **bold**. Please find our responses below the reviewer’s comment.

Sincerely,

Emily O’Brien, PhD, FAHA

Associate Professor

Duke Clinical Research Institute

Duke University School of Medicine

Department of Population Health Sciences

215 Morris Street, Suite 210

Durham, NC 27701

**REVIEWER #1 Comment:**

**Thank you again for the opportunity to review the revised manuscript.
The authors have done good job in reviewing the manuscript by addressing most comments. Overall, the comments are satisfactory. I have few concerns now as follows.
Comment number 3 in the previous revision is not satisfactory. How can COVID-19 be equated with trauma in accordance with ICD or DSM? Please be more concrete in justification.
In the manuscript, the authors mention that >80% of the samples were females and in results, the nurses have more PTSD symptoms. So, could it be because of the select sample? Please clarify.
Otherwise, the manuscript is good to go.
All the best.**

Author Response: Thank you for the opportunity to clarify this first point and add depth to our introduction. Per the reviewer’s suggestion, we have added the following languages and references.

“Prior research indicates that ‘helpers’ during human disasters often face psychological trauma (1). Analyses of post-9/11 experiences among first responders underscored the psychological risks for professionals exposed to suffering (2), with terms like compassion fatigue, secondary traumatic stress, and vicarious trauma used to describe such effects. This suggests that healthcare providers treating COVID-19 patients may also experience similar trauma, given their critical role in response to this crisis.”

Furthermore, we understand the reviewer's concern that our sample size was largely female and therefore there might be some confounding by gender and professional role. Our adjusted model should account for confounds such as gender but we recognize that can’t rule out residual confounds by other unmeasured factors. We have added the following language to the limitation section to address this.

“Moreover, although our adjusted model controlled for several important confounders such as gender, we cannot rule out the possibility of residual confounding from additional unmeasured factors.”
